# Reduced Tau protein expression is associated with frontotemporal degeneration with progranulin mutation

**DOI:** 10.1186/s40478-016-0345-0

**Published:** 2016-07-19

**Authors:** Anthony Papegaey, Sabiha Eddarkaoui, Vincent Deramecourt, Francisco-Jose Fernandez-Gomez, Pierre Pantano, Hélène Obriot, Camille Machala, Vincent Anquetil, Agnès Camuzat, Alexis Brice, Claude-Alain Maurage, Isabelle Le Ber, Charles Duyckaerts, Luc Buée, Nicolas Sergeant, Valérie Buée-Scherrer

**Affiliations:** University of Lille, Inserm, CHU-Lille, F-59000 Lille, France; Sorbonne Universités, UPMC Univ Paris 06, Hôpital Pitié-Salpêtrière, Paris, France; INSERM UMRS_1127, Hôpital Pitié-Salpêtrière, Paris, France; CNRS UMR_7225, Hôpital Pitié-Salpêtrière, Paris, France; AP-HP, Hôpital Pitié-Salpêtrière, Paris, France; ICM, Hôpital Pitié-Salpêtrière, Paris, France; Université Artois, Faculté Jean Perrin, F-62307 Lens, France; Inserm UMRS1172 – Alzheimer & Tauopathies, Faculty of Medecine-Research Pole, University of Lille, Place de Verdun, F-59045 Lille cedex, France

**Keywords:** Frontotemporal lobar degeneration, Tau protein, Progranulin, Synaptic impairment, Astrogliosis

## Abstract

Reduction of Tau protein expression was described in 2003 by Zhukareva et al. in a variant of frontotemporal lobar degeneration (FTLD) referred to as diagnosis of dementia lacking distinctive histopathology, then re-classified as FTLD with ubiquitin inclusions. However, the analysis of Tau expression in FTLD has not been reconsidered since then. Knowledge of the molecular basis of protein aggregates and genes that are mutated in the FTLD spectrum would enable to determine whether the “Tau-less” is a separate pathological entity or if it belongs to an existing subclass of FTLD. To address this question, we have analyzed Tau expression in the frontal brain areas from control, Alzheimer’s disease and FTLD cases, including FTLD- Tau (*MAPT)*, FTLD-TDP (sporadic, FTLD-TDP-*GRN*, FTLD-TDP-*C9ORF72*) and sporadic FTLD-FUS, using western blot and 2D-DIGE (Two-Dimensional fluorescence Difference Gel Electrophoresis) approaches. Surprisingly, we found that most of the FTLD-TDP-*GRN* brains are characterized by a huge reduction of Tau protein expression without any decrease in Tau mRNA levels. Interestingly, only cases affected by point mutations, rather than cases with total deletion of one *GRN* allele, seem to be affected by this reduction of Tau protein expression. Moreover, proteomic analysis highlighted correlations between reduced Tau protein level, synaptic impairment and massive reactive astrogliosis in these FTLD-*GRN* cases. Consistent with a recent study, our data also bring new insights regarding the role of progranulin in neurodegeneration by suggesting its involvement in lysosome and synaptic regulation. Together, our results demonstrate a strong association between progranulin deficiency and reduction of Tau protein expression that could lead to severe neuronal and glial dysfunctions. Our study also indicates that this FTLD-TDP-*GRN* subgroup could be part as a distinct entity of FTLD classification.

**Electronic supplementary material:**

The online version of this article (doi:10.1186/s40478-016-0345-0) contains supplementary material, which is available to authorized users.

## Introduction

Frontotemporal Lobar Degeneration (FTLD) accounts for 10 to 20 % of all demented cases. With an onset usually occurring between 45 and 64 years of age, FTLD represents the second common cause of dementia in the presenile age group (<65 years of age) [1]. FTLD is a clinical syndrome characterized by progressive deterioration in behavior, personality and/or language. Depending on the first and prevailing symptoms, there are three different clinical subtypes including the behavioral variant FTLD (bvFTLD) and two subtypes of primary progressive aphasia: progressive nonfluent aphasia (PNFA) and semantic dementia [2, 3]. In addition, movement disorder can also be observed in 10 to 15 % of FTLD cases (corticobasal syndrome, parkinsonism and/or amytrophic lateral sclerosis (ALS)) [4]. Given this phenotype variability, FTLD clinical diagnosis remains difficult and uneasy to establish with certainty [5]. However, genetics has allowed for a better stratification of FTLD spectrum. In fact, gene mutations also play an important role in FTLD with 30 to 50 % of patients reporting a positive family history of FTD and 10 to 15 % of patients corresponding to dominantly inherited form [6]. Firstly described are the *MAPT* mutations [7]. Mutations in the progranulin gene *GRN* were then found to be the most frequent mutations associated with FTLD [8, 9]. More recently, two studies demonstrated that expanded hexanucleotide GGGGCC repeats in a noncoding region of the chromosome 9 open reading frame 72 (*C9ORF72*) gene was responsible for a large proportion of both familial FTLD and ALS [10, 11]. Less frequently mutations in the valosin containing protein (*VCP*) gene or charged multivesicular body protein 2B (*CHMP2B*) gene are also found associated with FTLD [12, 13].

The definite diagnosis relies on neuropathological examination of the brain, the characteristics of these brain lesions and their molecular basis [14]. Indeed, as many neurodegenerative diseases, FTLD are characterized by the presence of protein aggregates in the affected brain regions. However, in contrast to the well-characterized nature of protein inclusions (Aβ plaques and neurofibrillary tangles) in Alzheimer’s disease (AD), proteinaceous aggregates in FTLD can be formed of different proteins [15]. Thus, approximatively 40 % of FTLD cases display aggregates made of abnormally and hyperphosphorylated Tau proteins and constitute the FTLD-Tau subclass. However, most of FTLD brains are negative for Tau inclusions and exhibit neuronal cytoplasmic and/or nuclear inclusions immunoreactive for transactive response DNA binding protein 43 (TDP-43) and constitute the FTLD-TDP subclass [16, 17]. This latter is subdivided into sporadic FTLD-TDP, FTLD-TDP-*GRN* (patients with mutations on *GRN*) and FTLD-TDP-*C9ORF72* (patients with mutations on *C9ORF72*) [8–11]. To a lesser extent, another protein called FUS (Fused in Sarcoma protein) is found in aggregates that are Tau and TDP-43 negative [18, 19]. This subclass is thus named FTLD-FUS. Finally, inclusions negative for Tau, TDP-43 or FUS are observed in rare cases of FTLD and associated with ubiquitin-proteasome system related proteins (FTLD-UPS) [20].

Prior to the discovery of the main molecular actors of FTLD, studies described a partial or total loss of soluble or physiological Tau protein expression in both grey and white matter [21, 22]. This loss of Tau was originally found in a subset of dementia called DLDH for Dementia Lacking Distinctive Histopathology (renamed later FTLD-ni for FTLD with no inclusion) [23]. In 2006, most of these cases were reclassified as FTLD-U (presenting with ubiquitin positive inclusions) [24]. However, additional investigation with specific regards to this loss of Tau expression has not been reported since Zhukareva et al. in 2003. With the progress in genetics and neuropathology of FTLD, the question of whether this reduction of Tau expression is seldom remains ill-defined. In this study, we used western blot analysis to investigate human brain Tau protein expression in Control, AD, FTLD-Tau, FTLD-TDP-*GRN*, FTLD-TDP-*C9ORF72*, sporadic FTLD-TDP and sporadic FTLD-FUS brains. Remarkably, we demonstrated a huge reduction of all six human brain Tau isoforms only in a subset of FTLD-TDP brains with mutation on the *GRN* gene. Thus, our data clearly suggest that these specific cases, referred to as FTLD-TDP-*GRN*lτ (lτ for low levels of Tau protein), could be part of the current classification as a distinct entity with more severe synaptic dysfunction and astrogliosis.

## Materials and methods

Frontal cortical brain tissues from Controls (*n* = 8), AD (*n* = 8), FTLD-Tau (*n* = 6), FTLD-TDP-*GRN* (*n* = 10), FTLD-TDP-*C9ORF72* (*n* = 10), sporadic FTLD-TDP (*n* = 8) and sporadic FTLD-FUS (*n* = 5) were provided from both Lille Neurobank and GIE NeuroCeb in Paris. The brain banks fulfill criteria from the French Law on biological resources including informed consent, ethics review committee and data protection (article L1243-4 du Code de la Santé publique, August 2007).

### Biochemical analysis

Frontal grey matter necropsic tissues (around 100 mg) were homogenized in UTS buffer (Urea 8 M, Thiourea 2 M, SDS 2 %) using a tissue grinder Potter-Elvehjem with a PTFE Pestle. The homogenate was further sonicated on ice and spun at 7500 × g during 10 min to remove tissue debris. The supernatant was kept at −80 °C until use. Protein amount was determined by Bradford protein assay, subsequently diluted in NuPAGE® lithium dodecyl sulfate (LDS) 4× sample buffer (glycerol 40 %, LDS 4 %, Ficoll 400 4 %, Triethanolamine chloride 800 mM, phenol red 0.025 % and Coomassie G250 0.025 %, EDTA disodium 2 mM, pH 7.6) supplemented with NuPAGE® sample reducing agents (Invitrogen) and loaded onto 4–12 % NuPAGE® Bis-Tris Novex Gels. Proteins were transferred on nitrocellulose membrane of 0.45 μM porosity (GE Lifesciences) using liquid transfer XCell II™ Blot Module, according to the manufacturer’s instructions (Invitrogen). After saturation for 30 min at room temperature with TNT (Tris 15 mM, pH 8, NaCl 140 mM, Tween 0.05 %) added with 5 % skimmed milk powder or 5 % BSA, membranes were rinsed three times 10 min with TNT and thereafter incubated with primary and secondary horseradish peroxidase-coupled antibodies. All primary antibodies and dilutions are listed in Table 1. The peroxidase activity was revealed using a chemiluminescence kit (ECL, GE Lifesciences) and an ImageQuant™ LAS4000 biomolecular imaging system (GE Lifesciences), according to the manufacturer’s instructions. Quantifications were performed using ImageJ 1.46 software (NIH Software).

**Table 1:** Antibodies used in this study

Name	Abbreviation	Epitope	Origin	Provider	Dilution	Reference
Tau						
Anti-total Tau (N-ter)	N-ter	First 19 aa in amino-terminal region	Rabbit	Home-made	1/10 000	[70]
Anti-total- Tau (Tau 5)	Tau 5	Middle region of Tau (aa 218–225)	Mouse	Invitrogen	1/2 000	[71]
Anti-total-Tau (C-ter)	C-ter	Last 15 aa in carboxy-terminal region	Rabbit	Home-made	1/10 000	[72]
Synaptic proteins						
α-synuclein	α-syn	Aa 15–123 of rat synuclein-1	Mouse	BD Labsciences	1/500	[73]
Post-synaptic density 95	PSD-95	Human PSD-95	Rabbit	Cell Signaling	1/1000	[74]
Munc-18	Munc-18	Aa 577–594 of rat Munc-18	Rabbit	Sigma	1/10 000	[75]
Synaptophysine	SYP	Aa 221–313 of human SYP	Mouse	Santa Cruz	1/10 000	[76]
Astrocytic proteins						
Glutamine synthetase	GS	Aa 250–350 of Human GS	Rabbit	Abcam	1/10 000	N/A
Glial Fibrillary Acidic Protein	GFAP	Bovin GFAP FL	Mouse	Santa Cruz	1/1000	[77]
Others						
β-actin	Actin	N-ter	Mouse	Sigma-Aldrich	1/10 000	N/A
Neuron Specific Enolase	NSE	Aa 269–286 of Human NSE	Rabbit	Enzo Life Science	1/50 000	N/A
Aconitase		Bovine heart mitochondria	Mouse	Abcam	1/1000	[78]
Histone H3	H3	C-terminus of human H3	Rabbit	Millipore	1/10 000	[79]

For each antibody, the full name, abbreviation, recognized sequence, origin, provider, dilution and literature reference are given. *N/A* Not Available

### Sample preparation for two-dimensional differential gel electrophoresis (2D-DIGE)

Frozen UTS brain samples (a total of 1.5 mg of protein for each condition) was unfrozen on ice and proteins were precipitated using chloroform/methanol precipitation [25]. The protein-dried pellet was resuspended in UTC buffer (Urea 8 M, Thiourea 2 M supplemented with 4 % CHAPS) and kept at −80 °C until use. Protein concentration was measured using Quick-Start Bradford Dye Reagent (Bio-Rad) and sample quality was evaluated by loading 15 μg of proteins onto 4–12 % NuPAGE® Bis-Tris Novex Gels and stained with Coomassie R-250 (Biorad).

### 2D-DIGE

The 2D-DIGE was performed as previously described [25]. Briefly, 50 μg of protein was covalently coupled with 400 pmol of cyanine dyes diluted in dimethylformamide, according to the manufacturer’s instructions (CyDIGE, GE Lifesciences). Each sample was labeled with either Cy3 or Cy5 fluorescent dyes (GE Lifesciences) and kept for 1 h at 4 °C in darkness. Cross-labeling with either Cy3 or Cy5 dyes was performed in order to avoid a preferential coupling of one cyanine to a sample. A pool of both samples containing equal amount of protein (50 μg in total) was labeled with Cy2 fluorescent dye and used as internal standard in accordance with the manufacturer’s instructions (GE Lifesciences). Finally, the internal standard labeled with Cy2 and the samples labeled with either Cy3 or Cy5 were pooled and the final volume was adjusted to 350 μL by the addition of rehydration buffer [Urea 8 M, Thiourea 2 M, CHAPS 2 %, Destreak reagent 1.1 % (GE Lifesciences), IPG buffer pH 3–11 1.2 % (GE Lifesciences), bromophenol blue 0.01 %]. Samples were prepared in quadruplicate and loaded onto four independent IPG strips. Eighteen cm long linear pH gradient of 3–11 IPG strips (GE Lifesciences) were rehydrated overnight with the samples in a rehydration cassette recovered with mineral oil. Excess or mineral oil was discarded and isoelectrofocalisation was achieved using IPGphor isoelectric focusing apparatus (GE Lifesciences). A seven steps procedure was applied with the following conditions: 150 V for 1 h, 200 V for 5 h, 200 V to 500 V step gradient for 2 h, 500 to 1000 V step gradient for 2 h, 1000 V to 4000 V gradient for 2 h, and finally 8000 V gradient for 2 h. Current was limited to 50 μA per strip. Strips were then equilibrated in equilibration buffer (Urea 6 M, SDS 2 %, Glycerol 30 %, Tris–HCl 50 mM, pH 8.6) with successively 1 % DTT (dithiothreitol) and 4.7 % iodoacetamide for 15 min. Proteins were then separated in the second dimension on 1 mm-thick 12 % SDS-PAGE gels in an ETTAN DALTSix system (GE Lifesciences). Gels were run at 2.5 W per gel overnight. Fluorescently labeled protein spots were visualized using a Typhon FLA 9500 imager (GE Lifesciences). Gels were scanned at 200 μm resolution and images were exported for further analysis using SameSpots (TotalLab) software.

### Data analysis

Spot detection and relative quantification of spot intensity were analyzed using 2-DIGE analysis software package SameSpots (TotalLab). One-way ANOVA statistical test was applied and expression change was considered as significant with an exact *p*-value below 0.05. Normalization across all gels was performed using the internal standard.

### Preparative 2D gels

In order to identify proteins of interest, two preparative 2D-gels with respectively 500 μg of brain protein of each condition were performed. After electrophoresis, gels were fixed in ethanol 30 %, orthophosphoric acid (OPA) 2 % overnight. Following washing in OPA 2 %, gels were incubated 30 min in pre-coloration buffer (ethanol 18 %, OPA 2 % and ammonium sulfate 0.9 M) before Coomassie blue staining (Brillant Blue G-250, Bio-Rad) for 48 h.

### Trypsin digestion, mass spectrometry and protein identification

Spot labelling shown to be significantly different between two conditions after SameSpots analyses was manually excised from preparative gels. Each separate spot was incubated in DTT 10 mM and alkylated (iodoacetamide 55 mM) before trypsin digestion (Promega) overnight at 37 °C, according to the manufacturer’s instructions. Supernatants, containing digested peptides, were dried using centrifuge vacuum (Concentrator 5301, Eppendorf) and resuspended in ultra-pure water supplemented with trifluoroacetic acid (TFA) 0,1 %. The resulting peptide mixture was spotted onto a MALDI plate with freshly dissolved α-cyano-4-hydroxycinnaminic acid (10 mg/ml in acetonitrile 50 %, TFA 0.1 %). Mass spectrometry was achieved with a MALDI-TOF-TOF Autoflex Speed (Bruker Daltonics). MS and MS/MS data were analyzed with BioTools software and peptides sequences were analyzed with Mascot (http://www.matrixscience.com/). A mascot score above 61 was considered significant for protein identification.

### mRNA extraction and quantitative real-time polymerase chain reaction (RT-qPCR) analysis

Total RNA was extracted from the tissue of the frontal cortex and purified using the RNeasy Lipid Tissue Mini Kit (Qiagen) following the manufacturer’s instructions. For each RNA sample, integrity (RIN, RNA Integrity Number) was assessed on 2100 bioanalyzer (Agilent Technologies, Waldbronn, Germany) using the RNA 6000 nano kit according to the manufacturer protocol.

One microgram of total RNA was reverse-transcribed using Applied Biosystems High Capactiy cDNA reverse transcription kit. RT-qPCR analysis was performed using an Applied Biosystems Prism 7900 SYBR Green PCR Master Mix. The amplification conditions were as follows: initial step of 10 min at 95 °C, followed by 45 cycles of a 2-step PCR consisting of a 95 °C denaturing step for 15 s followed by a 60 °C extension step for 25 s. Primers used were: Tau 5’UTR 5’ACAGCCACCTTCTCCTCCTC3’ and 5’ GATCTTCCATCACTTCGAACTCC3’; Tau E11-12 5’ACCAGTTGACCTGAGCAAGG3’ and 5’ AGGGACGTGGGTGATATTGT3’ and RPLP0 5’GCAATGTTGCCAGTGTCTG3’ and 5’ GCCTTGACCTTTTCAGCAA3’. Amplifications were carried out in triplicate and the relative expression of target genes was determined by the ΔΔC_T_ method [26].

### Statistical analysis

For western blot and RT-qPCR statistical analyses, the non-parametric Mann–Whitney test or the Kruskall-Wallis test were performed. All statistical analyses were performed using the GraphPad Prism 6 program (GraphPad Software) and statistical significance was set at * *p* < 0.05, **p < 0.01, ***p < 0.001, ****p < 0.0001.

## Results

### Neuropathology

Neuropathological assessment of all cases was performed in the departments of anatomo-pathology of CHU-Lille and Hôpital Pitié-Salpêtrière. Detailed information on pathology and demographic data are summarized in Table 2.

**Table 2:** Demographic data on studied cases

Genetic diagnosis	Cases	Neuropathology	Age (yr)	Sex	PMD (hr)	RIN (a.u)	Fixed hemibrain (g)	Genetic variant
Controls	1		73	M	10	6,6	508	
	2		84	M	15,5	5,3	N/A	
	3		70	M	31	5,6	765	
	4		76	F	28	4,8	580	
	5		86	F	N/A	5,1	540	
	6		79	M	N/A	6,3	675	
	7		69	M	6	6,9	632	
	8		60	F	28	6,8	788	
FTLD, sporadic	9	FTLD-TDP Type A	84	M	25	4,4	614	
	10	FTLD-TDP Type A	42	M	37	5,7	684	
	11	FTLD-TDP Type C	86	M	44	4,8	504	
	12	FTLD-TDP	67	F	22	6,3	495	
	13	FTLD-TDP Type C	68	M	N/A	2,5	430	
	14	FTLD-TDP Type C	72	M	16	5,8	452	
	15	FTLD-TDP Type B	53	M	5	8	600	
	16	FTLD-TDP Type B	77	M	17	5,5	378	
	17	FTLD-FUS	35	M	64	5,5	N/A	
	18	FTLD-FUS	59	M	30	6,6	430	
	19	FTLD-FUS	44	M	11	5,7	504	
	20	FTLD-FUS	35	F	17	5,9	495	
	21	FTLD-FUS	54	M	18	4,8	424	
FTLD, *GRN*	22	FTLD-TDP Type A	71	M	23	3	556	c.813_816del
	23	FTLD-TDP Type A	69	F	39	3,8	370	c.1494_1498del
	24	FTLD-TDP Type A	60	F	N/A	3,7	200	c.1494_1498del
	25	FTLD-TDP Type A	65	F	N/A	5,9	N/A	c.619dup
	26	FTLD-TDP Type A	67	M	22	3,4	608	c.813_816del
	27	FTLD-TDP Type A	69	M	18,5	7	166	c.1494_1498del
	28	FTLD-TDP Type A	78	F	20	4	420	N/A
	29	FTLD-TDP Type A	75	M	21	5,3	419	c.1157G > A
	30	FTLD-TDP Type A	73	M	10	5,6	456	Complete deletion
	31	FTLD-TDP Type A	86	F	5,5	6,8	388	Complete deletion
FTLD, *C9ORF72*	32	FTLD-TDP	59	M	51	6,4	N/A	
	33	FTLD-TDP Type B	42	M	10	3,2	N/A	
	34	FTLD-TDP Type B	40	F	48	4,5	N/A	
	35	FTLD-TDP Type B	63	M	13	5,2	762	
	36	FTLD-TDP	90	M	40	4,4	N/A	
	37	FTLD-TDP	62	M	N/A	5,1	541	
	38	FTLD-TDP Type B	69	M	8,5	4,4	N/A	
	39	FTLD-TDP Type B	65	M	20	5,3	400	
	40	FTLD-TDP Type B	62	F	5,5	7,6	258	
	41	FTLD-TDP Type B	59	M	8,5	6	438	
FTLD, *MAPT*	42	FTLD-Tau	48	F	44,5	6,1	N/A	P301L
	43	FTLD-Tau	54	F	N/A	7,8	N/A	S305S
	44	FTLD-Tau	43	M	6	4,8	N/A	P301L
	45	FTLD-Tau	65	F	30,5	3,4	315	P301L
	46	FTLD-Tau	66	M	30	5,9	N/A	P301L
	47	FTLD-Tau	85	F	21	5	360	P332S
AD	48	AD	79	F	48	4,7	474	
	59	AD	73	F	26	4,7	460	
	50	AD	55	F	N/A	4,8	416	
	51	AD	75	M	30	4,1	529	
	52	AD	74	M	10	3	N/A	
	53	AD	63	M	18	6,1	366	
	54	AD	61	M	23	7	414	
	55	AD	62	M	10	6	435	

*AD* Alzheimer’s disease. *C9ORF72*, chromosome 9 open reading frame 72, *FTLD* FrontoTemporal Lobar Degeneration, *GRN*, progranulin, *MAPT* microtubule-associated protein tau, *PMD* postmortem delay, *RIN* RNA Integrity Number, *sp*, sporadic cases. *a.u* arbitrary unit, *N/A* Not Available

### Reduction of Tau protein expression is observed in FTLD-TDP brains associated with GRN gene mutation without Tau mRNA decrease

**Fig. 1:**
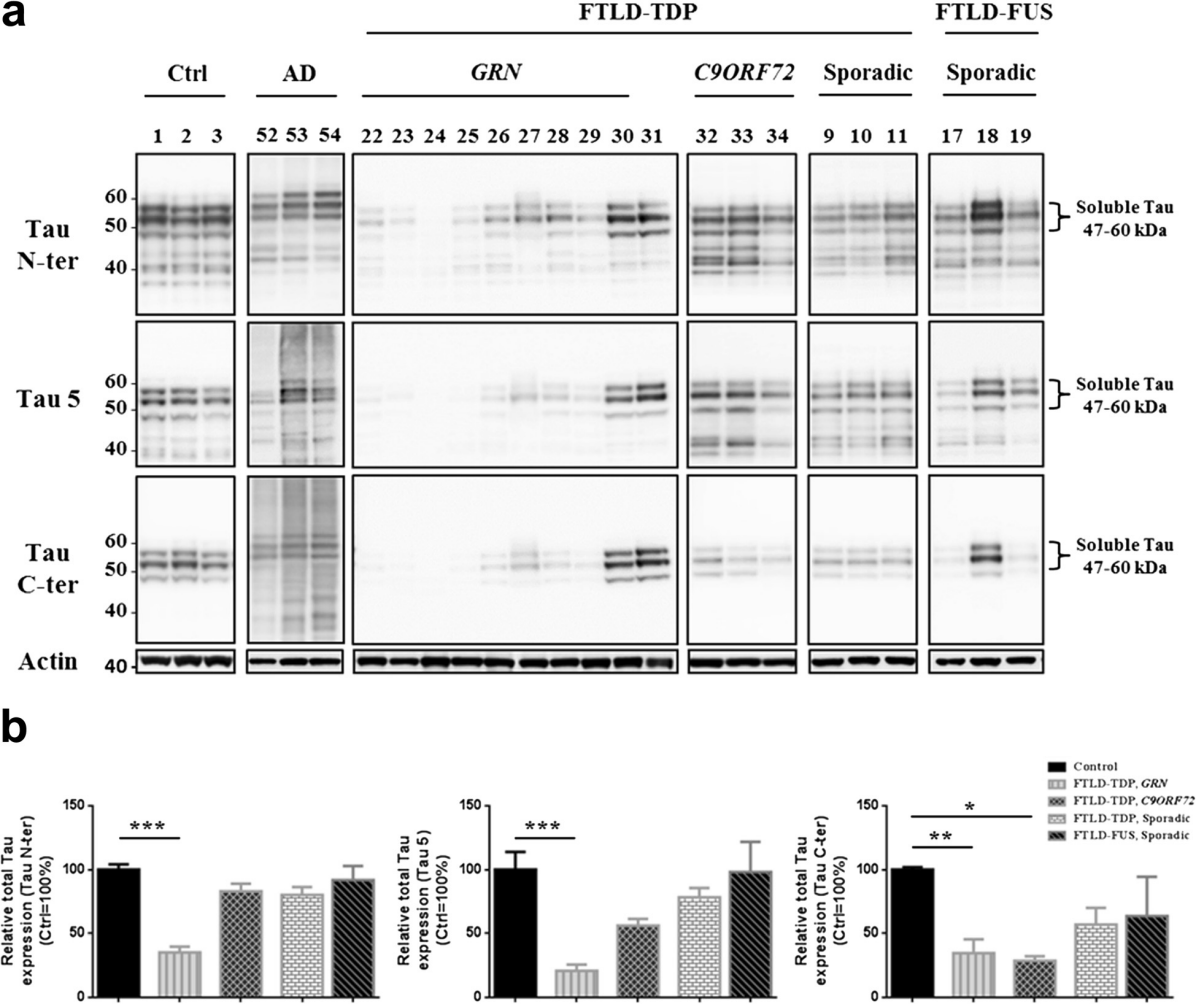
Reduction of Tau protein expression in FTLD brains. **a** Western blot analysis of soluble Tau protein expression in control, AD and FTLD brains using antibodies targeting total Tau independently of any post-translational modification (N-ter, Tau 5 and C-ter). Are shown representative data from FTLD-TDP-*GRN* (*n* = 10), FTLD-TDP*-C9ORF72* (*n* = 10), sporadic FTLD-TDP (*n* = 8), sporadic FTLD-FUS (*n* = 5), AD (*n* = 8) and control brains (*n* = 8). **b** Total Tau levels were quantified and normalized to a pool containing same protein amount of each control used in this study. Both full-length and truncated Tau species were considered for the quantification. Actin was used as loading control. Results are expressed as means ± SEM. For statistical analysis the Kruskal-Wallis test was used (*p < 0.05, **p < 0.01; ***p < 0.001). SEM: standard error of the mean; kDa: kiloDalton

Tau protein expression was studied in all cases. We first checked by western-blotting, if there was any Tau pathology in these brains, since it has been described in AD, and a subset of FTLD-TDP patients [27, 28]. Lack of phospho-Tau immunoreactivity was a condition to exclude Tau pathology and therefore any FTLD-Tau as compared with FTLD-*MAPT* and AD (data not shown). Thereafter, Tau expression was investigated by using antibodies targeting Tau protein independently of its phosphorylation state to evaluate total Tau protein level. These well-characterized antibodies either target the amino-terminal (Tau N-ter), the median (Tau 5) or carboxy-terminal epitope of Tau (Tau C-ter). In adult human brain, six Tau isoforms are expressed from *MAPT* gene through alternative mRNA splicing [28]. These six Tau isoforms give rise to a unique biochemical signature made of three bands (Fig. 1a, Control patients 1 to 3). Thus, western blot analysis highlighted significant decrease of all six Tau isoforms in eight FTLD-TDP brains compared to control, AD and other FTLD brains (patients 22 to 29, Fig. 1a). Interestingly, this decrease is observed with all three Tau antibodies suggesting that Tau holoprotein isoform expression is impaired (Fig. 1a, compare patient 25 with patient 33). More interestingly, this reduction of Tau protein expression is restricted to FTLD-TDP brains associated with mutations on the *GRN* gene (Fig. 1a and b) and not associated with other FTLD-related gene mutations. Indeed, Tau protein expression in FTLD-TDP-*C9ORF72*, sporadic FTLD-TDP or FTLD-FUS patients is rather homogeneous from one patient to another with each antibody (Fig. 1a). Consequently to these results, *GRN* cases with reduced Tau protein levels were designated as FTLD-TDP-*GRN*lτ and other FTLD-TDP cases with conserved Tau protein expression as FTLD-TDPτ.

This reduced Tau protein level could result from a lower transcription of *MAPT* gene in these brains. However, RT-qPCR using primers targeting constitutively Tau mRNA encoded sequences [5’ UTR and exons 11–12 (E11-12)] revealed no significant decrease in total Tau mRNA level whatever the neuropathological group considered (Additional file 1: Figure S1). Therefore, consistent with previous data published in 2001 [21], these data confirm a reduction in Tau protein expression that cannot be explained by a *MAPT* gene trancription modification. But more interestingly, herein we show that this decrease in Tau protein expression is restricted to patients with *GRN* mutations.

### FTLD-TDP-GRNlτ brains display more astrogliosis and neuronal dysfunction compared to other FTLD-TDPτ brains

**Fig. 2:**
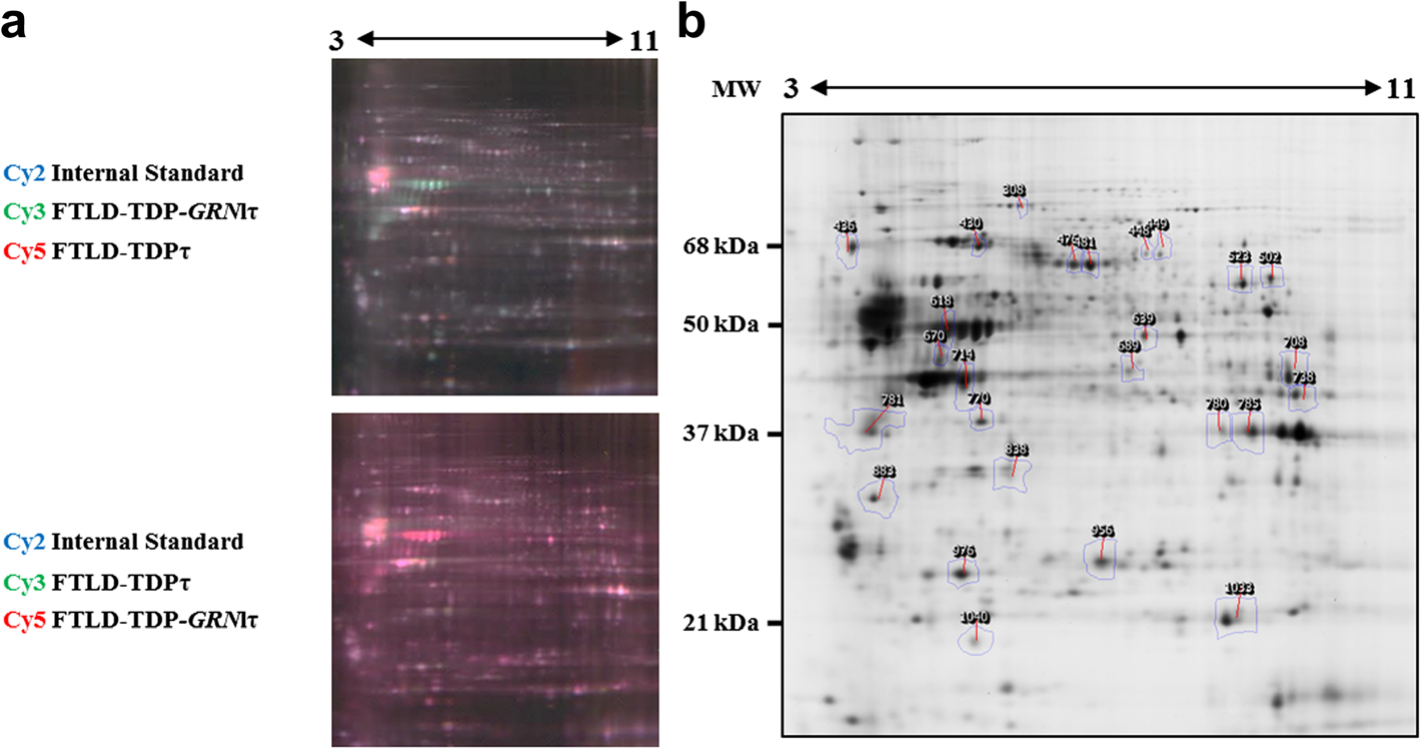
2D-DIGE analysis of FTLD-TDP-*GRN*lτ and FTLD-TDPτ cases. Analysis of 2D-DIGE gels was performed using TotalLab SameSpot software. **a** Overlay of 2D-DIGE images with the two possible combinations. In the upper panel, FTLD-TDP-*GRN*lτ pool is labeled with Cy3 (green) and FTLD-TDPτ brains with Cy5 (red). In the lower panel, FTLD-TDP-*GRN*lτ pool is labeled with Cy5 (red) and FTLD-TDPτ brains with Cy3 (green). In both combination, the internal standard is labeled with Cy2 (blue). **b** 2D-DIGE map of proteins which are deregulated in FTLD-TDP-*GRN*lτ samples compared to FTLD-TDPτ samples. Spots of interest (numbers) are listed and described in Table 3. kDa: kiloDalton; MW: molecular weight

Since Tau protein level is reduced but not mRNA, we investigated if other proteins could be modified. We therefore performed a quantitative proteomic analysis using 2D-DIGE to evaluate any dysregulation of other protein expression. For this purpose, proteomes of FTLD-TDPτ brains (*n* = 3, cases 14, 15 and 16) and FTLD-TDP-*GRN*lτ (*n* = 3, cases 22, 24 and 25) were compared. Following bioinformatics assisted analysis of 2D-DIGE gels (*n* = 4), 26 protein spots with significant differential level of expression between FTLD-TDP-*GRN*lτ and FTLD-TDPτ brains were isolated for further identification (Fig. 2a, b; Table 3). According to the mass spectrometry analysis, 20 distinct proteins including 6 isovariants of the same protein were identified. Among the 20 proteins identified with a significant mascot score (>61), the amount of seven proteins decreased while that of 13 increased in FTLD-TDP-*GRN*lτ (Table 3). Eleven proteins which intensity varies belong to proteins involved in metabolism (Table 3). Stress-related protein HSP-70.1 and structural proteins such as Gelsolin and Neurofilament light chain showed an increased expression (Table 3). A decrease of *UCHL1* (spot 976, −1.3 fold change), a neuronal enzyme involved in ubiquitinated proteins processing, was also found (Table 3). Interestingly, decrease and modification in proteins involved in synaptic function (*STXB1*, *DPYL2* and *GLNA* gene product in spot 448, 481, 689 with −1.3, −1.3 and −1.2 fold change, respectively) were observed suggesting a stronger synaptic impairment in the FTLD-TDP-*GRN*lτ group (Table 3). Regarding glial cells, a decrease in glutamine synthetase (GS; astrocytic enzyme involved in glutamate metabolism) was observed with a −1.2 fold change, whereas the highest fold change (+3.2) was related to four spots corresponding to GFAP (Table 3). Taken together, these data demonstrate a strong correlation between reduction of Tau protein expression, astrocytic and synaptic dysfunctions. Therefore, these proteomic data highlight quantitative dysregulation of protein expression other than Tau proteins in the brain from patients with FTLD-TDP bearing *GRN* mutations.

**Table 3:** Proteins differentially expressed between FTLD-TDP-*GRN*lτ and FTLD-TDPτ

Spot n°	Protein name	Accession No.	Gene name	*p-*value	Fold change	Theoretical pI/MW	Apparent pI/MW	Mascot score	% sequence coverage
308	Gelsolin	P06396	*GSN*	0.001	+1.4	5.9/85	5.9/85	77	1.4
436	Neurofilament light polypeptide	P07196	*NEFL*	4.57E-04	- 1.5	4.5/61.5	4.5/61.5	202	34.3
Metabolism related proteins									
780	Glyceraldehyde-3-phosphate dehydrogenase (2)	P04406	*GAPDH*	0.001	+1.3	8.6/36	8.4/36	63.5	21.2
1040	Ferritin light subunit	P02792	*FTL*	3.67E-06	+2.2	5.4/20	5.4/20	354.5	30.3
738	Fructose 1.6 biphosphate aldolase	P04075	*ALDOA*	0.0037	+1.2	9.2/39.4	9.2/39.4	81	33.5
639	Alpha-enolase	P06733	*ENO1*	1.57E-04	+1.9	7.7/47.1	7.7/47.1	178	44.5
1033	Phosphatidylethanolamine-binding protein 1	P30086	*PEBP1*	0.007	+1.3	7.4/21	8.4/21	101	56.7
956	Peroxiredoxin 6	P30041	*PRDX6*	1.94E-05	+1.9	6.3/25	7.0/25	147	52.2
523	Pyruvate Kinase M (2)	P14618	*PKM*	0.019	+1.2	9.0/60	8.4/60	93.9	31.8
708	Phosphoglycerate kinase 1	P00558	*PGK1*	0.023	+1.2	9.2/45	9.2/45	95.2	29.5
770	N(G).N(G)-dimethylarginine dimethylaminohydrolase 1	O94760	*DDAH1*	4.36E-04	+1.3	5.5/31.1	5.8/43	121	44.6
838	Guanine nucleotide-binding protein 1	P62873	*GNB1*	7.02E-05	- 1.6	5.6/37	5.6/37	117	42.9
714	Creatine Kinase B	P12277	*CKB*	0.004	- 1.2	5.2/42.6	5.6/42.6	206	54.9
Astrocytic related proteins									
618	Glial fibrillary acidic protein (3)	P14136	*GFAP*	2.43E-06	+3.2	5.3/49.8	5.5/49.8	287	56.4
689	Glutamine synthetase	P15104	*GLUL*	0.003	- 1.2	6.5/42	7.2/42	69.4	16.4
Synaptic related proteins									
476	Dihydropyrimidinase-related protein 2 (2)	Q16555	*DPYSL2*	4.62E-04	- 1.3	5.9/62.3	6.6/62.3	274	50.1
448	Syntaxin-binding protein 1 (2)	P61764	*STXB1*	3.70E-04	- 1.3	6.5/67.5	7.6/60	138	22.6
Other									
883	Annexin 5	P08758	*ANXA5*	1.75E-04	+1.5	4.8/35.9	4.8/35.9	188	45.6
976	Ubiquitin carboxyl-terminal hydrolase isoenzyme L1	P09936	*UCHL1*	0.003	- 1.3	5.2/25	5.2/25	85.8	47.1
430	Heat shock 70 kDa protein 1A	P0DMV8	*HSPA1A*	0.002	+1.3	5.4/70	5.4/60	110	27.9

Data obtained from Samespot software are presented for each spot of interest: spot number, *p*-value, fold change (FTLD-TDP-*GRN*lτ vs FTLD-TDPτ), experimental molecular weight (MW) and isoelectric point (pI). According to mass spectrometry identification of each protein, table also gives: the protein full name, accession number, gene name, mascot score, sequence coverage (%), and the theoretical molecular weight (MW) and pI of the non-modified protein. A mascot score above 61 was considered as significant for protein identification. Difference between theoretical and experimental molecular weight or pI is underlined. Number of isovariants for each protein spot is indicated with the protein name (see parenthesis)

### Proteomic results validation in brain samples highlight specific dysregulation in FTLD-TDP-GRNlτ brains

**Fig. 3:**
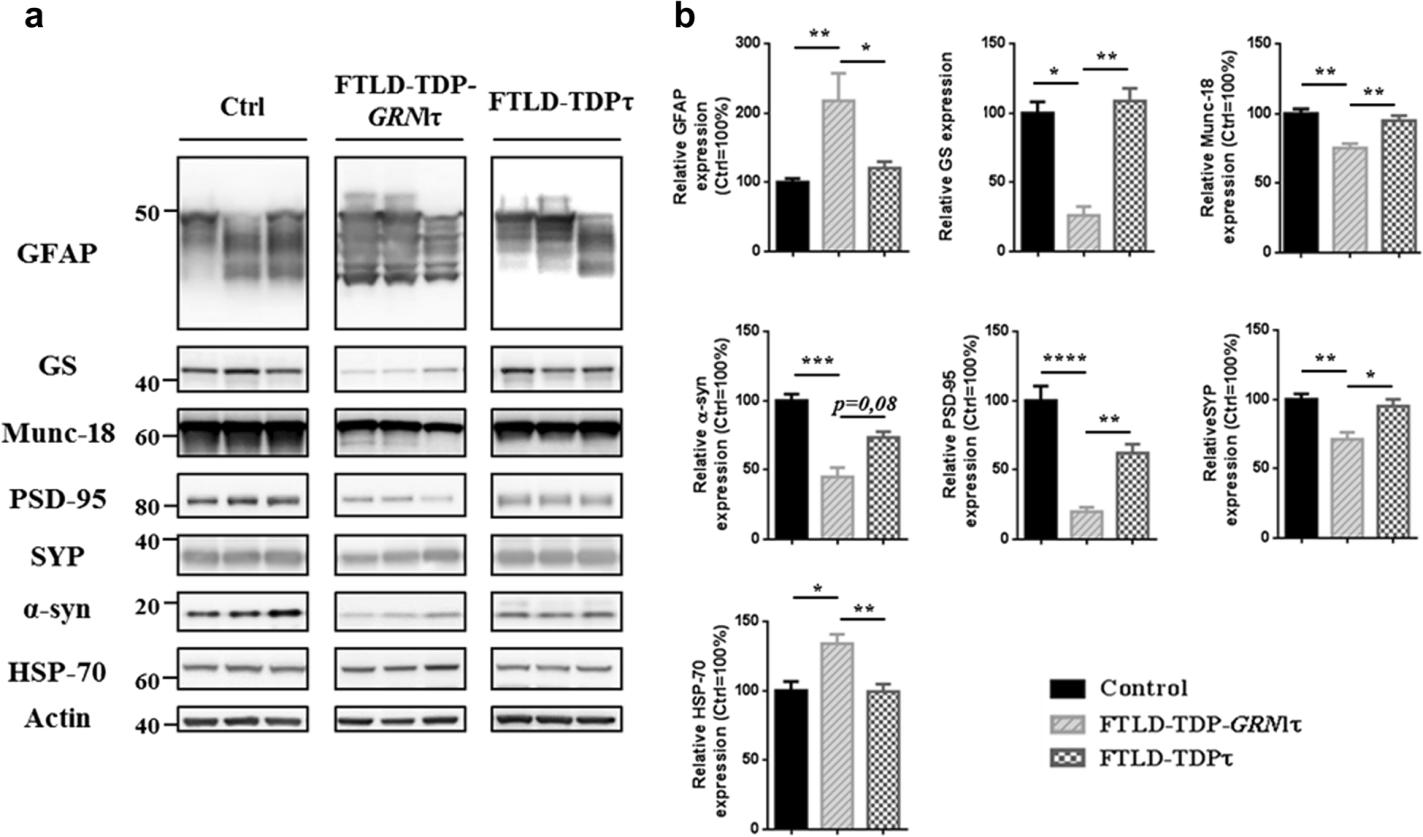
Biochemical validation of 2D-DIGE results in control, FTLD-TDP-*GRN*lτ and FTLD-TDPτ brain samples. **a** Western blot analysis of synaptic [PSD-95, *α*-synuclein (*α*-syn), Munc-18 and Synaptophysin (SYP)], astrocytic [GFAP and Glutamine Synthetase (GS)], and stress (HSP-70) related protein level in control, FTLD-TDP-*GRN*lτ and FTLD-TDPτ brain samples. Representative data from FTLD-TDP-*GRN*lτ (*n* = 8), FTLD-TDPτ (*n* = 20) and control brains (*n* = 8) are presented. **b** Protein levels were quantified and normalized to a pool containing same protein amount of each control used in this study. Actin was used as loading control. Results are expressed as means ± SEM. For statistical analysis the Kruskal-Wallis test was used (*p < 0.05; **p < 0.01; ***p < 0,001; ****p < 0,0001). SEM: standard error of the mean

To validate these proteomic results found in a subset of patients, we therefore undertook an analysis of dysregulated neuronal and astrocytic proteins in all brain samples (FTLD-TDP-*GRN*lτ, FTLD-TDPτ and control) using western blot analysis. We first confirmed an increase in HSP-70 protein level in FTLD-TDP-*GRN*lτ cases (Fig. 3a, b). Very strikingly, we observed as in 2D-DIGE, an upsurge in GFAP expression in FTLD-TDP-*GRN*lτ group in comparison with both control and other FTLD-TDP cases (Fig. 3a, b). Noteworthy, GS was found to be dramatically decreased (Fig. 3a, b). With regards to synaptic proteins, several synaptic markers were decreased including α-synuclein and PSD-95 (Fig. 3a, b). These dysregulations found in FTLD-TDP-*GRN*lτ brains could be the reflect of a global proteome deterioration in these samples. We thus tested the level of several proteins such as Neuronal Specific Enolase (NSE), Aconitase, Histone H3 and Neurofilaments. Their levels remain unchanged among the different FTLD subclasses (Additional file 2: Figure S2). Finally, it is also worth noting that among FTLD patients we could not find any correlation between Tau protein decrease, macroscopic atrophy, post-mortem delay (PMD) and RNA Integrity Number (RIN) (Table 2, Additional file 3: Figure S3a, b, c respectively). All these results provide further evidence that specific dysregulations affect FTLD-TDP-*GRN*lτ patients such as dramatic synaptic impairment and massive reactive astrogliosis.

## Discussion

For the first time since Zhukareva’s studies, our data clearly demonstrate that the reduced Tau protein expression is restricted to FTLD-TDP brains with mutations on *GRN* gene. Although several FTLD brains display a lower Tau protein level with Tau C-ter antibody, the labelling obtained with N-ter and Tau 5 shows a relative conservation of Tau protein expression suggesting a preferential degradation of Tau at the C-terminal part in these cases. In contrast, FTLD-TDP-*GRN*lτ brains exhibit reduced Tau levels with all Tau antibodies tested.

Consistent with previous studies, reduction of Tau protein expression is unlikely to result from extensive neuronal loss as demonstrated by the preserved expression of several specific neuronal proteins [21, 22]. Moreover, we could not find any correlation between reduced Tau level and PMD, RIN or cortical atrophy. Finally, downregulation of *MAPT* transcription does not appear to be responsible for this decrease in Tau since mRNA level remains unchanged in these FTLD-TDP-*GRN*lτ brains. Therefore, reduction of Tau protein might rather result from post-transcriptional dysregulations.

TDP-43, the main constituent of aggregates found in FTLD-TDP-*GRN*lτ cases, is involved in RNA metabolism and especially in mRNA transport and stability through 3’UTR binding of targeted transcripts (see [29–31] for review). Notably, a recent study showed that loss of TDP-43 function impairs microtubule-dependent transport of mRNA granules towards distal neuronal compartment [32]. Regarding axonal translation of Tau [33], loss of TDP-43 function may lead to deficient Tau protein translation. Nevertheless, this hypothesis suggests specific pathophysiological process in FTLD-TDP-*GRN*lτ when compared to other FTLD-TDP cases that do not display change in Tau protein level.

MicroRNAs (miRNAs) play a key role in both normal aging and neurodegenerative diseases (see [34, 35] for review). Interestingly, studies have reported that different miRNA are able to modulate Tau metabolism [36, 37]. Among them, miR-219 is particularly interesting since it modulates Tau protein translation with relatively low influence on total Tau mRNA level. Consistent with this study, it is worth noting that TDP-43 is also involved in miRNA biogenesis [38], suggesting that specific miRNA deregulation could lead to a reduction of Tau mRNA translation in FTLD-TDP-*GRN*lτ brains. Finally, emerging evidences indicate that Tau is physiologically released into extracellular space through multiple mechanisms such as multivesicular body and ectosome secretion [39]. It could therefore be interesting to evaluate Tau protein level in cerebrospinal fluid to see if an increase in Tau secretion participates to this reduction of Tau protein expression.

All FTLD-TDP-*GRN*lτ cases display mutation on the *GRN* gene. It is well established that mutations on *GRN* gene induce haploinsufficiency with approximatively 50 % reduction in mRNA levels and 33 % in protein level [8]. However, how progranulin haploinsuffiency leads to neurodegeneration is still unclear, in part due to the lack of progranulin-deficient models recapitulating FTLD hallmarks. Progranulin is a secreted protein widely expressed throughout the body that exerts numerous functions during development, tumor proliferation and inflammation (see [40, 41] for review). In adult brain, progranulin is mostly found in neurons and activated microglia [42] where it regulates neurite outgrowth [43], synapse biology [44], stress response [45] and lysosomal function [46]. All these data suggest a strong role of progranulin in neurodegenerative diseases but how can we relate the reduction of Tau with *GRN* mutations? Depending on the mutation, we observed very distinct phenotype between cases. Indeed, cases affected by a total deletion of one *GRN* allele do not display any decrease in Tau expression whereas other point mutations are associated with a huge reduction of all six isoforms. This result is remarkable and suggests for the first time that different mutations can induce distinct phenotype and not only haploinsufficiency. Indeed, homozygous deletion of *GRN* does not lead to FTLD-TDP but to another disorder called Neuronal Ceroid Lipofuscinosis (NCL) which is characterized by lysosomal dysfunction [46]. Thus, a recent study has demonstrated that specific granulins expression, resulting from progranulin extracellular cleavage, could have toxic effect [47]. These point mutations could lead to modified mRNA leading to the production of toxic granulins. However, the lack of information on the different granulins, and their functions are still unknown and the relationship with Tau metabolism, if any, remains to be experimentally established.

Reduction of Tau protein expression in FTLD-TDP-*GRN*lτ brains is intriguing since Tau has essential functions in neuron. Indeed, Tau protein is a microtubule associated protein (MAP) which mainly distributes into axons [48] and was originally described as a protein regulating the assembly and stabilization of microtubules [49, 50], therefore modulating axonal transport [51]. However, recent studies have highlighted a role for Tau in synaptic [52, 53] and nuclear compartments [54, 55]. Although initial studies showed that tau-knockout mice develop no evident pathology, probably through MAP1A compensatory effect [56], recent studies have revealed several pathological modifications in these knockout mice suggesting that Tau is essential for neuronal activity [57], iron export [58], neurogenesis [59] and both long-term depression and long-term potentiation [60, 61]. Regarding our results, it would not be surprising that decrease in Tau protein expression leads to neuronal dysfunction.

This hypothesis is strengthened by our 2D-DIGE analysis and biochemical validation, demonstrating that expression of several neuronal proteins is either up- or down-regulated. Indeed, both pre- and post-synaptic proteins such as PSD-95, Munc-18, α-synuclein, synaptophysin and syntaxin-binding protein 1 are highly reduced in FTLD-TDP-*GRN*lτ brains in comparison to control and FTLD-TDPτ brains. It’s interesting to note that a very recent study has described a link between synaptic dysfunction and progranulin deficiency [62]. Indeed, progranulin deficiency is able to induce synaptic pruning through lysosome dysfunctions and complement activation. It could explain, in part, the dramatic synaptic loss we found in FTLD-TDP-*GRN*lτ brains, in whom progranulin levels are very low. Finally, regarding downregulation of dihydropyriminidase-related protein 2 (DPYSL2), also called collapsin response mediator protein-2 (CRMP2), it should be noted that this protein serves important functions in synaptic plasticity. Moreover, CRMP2 and Tau are both high-abundance microtubule-associated proteins, and overlap in terms of functional regulation [63]. All these data demonstrate that synaptic functions are impaired in these FTLD-TDP-*GRN*lτ brains.

In parallel with these neuronal dysfunctions, an increase in GFAP expression is also observed in FTLD-TDP-*GRN*lτ brains. GFAP belongs to intermediate filaments and is expressed mostly in astrocytes. These glial cells are complex highly differentiated cells that perform numerous essential functions in central nervous system (CNS), such as synaptic function and plasticity and maintenance of the neuronal microenvironment homeostasis [64]. Astrocytes respond to various forms of CNS injury such as infections, ischemia or neurodegenerative diseases through a process referred to as reactive astrogliosis and often characterized by an increase in GFAP expression [65]. Although a mild to moderate reactive astrogliosis represents a protective mechanism, severe astrogliosis could lead to functional defects including alteration of astrocyte ability to control neuronal microenvironment homeostasis [66, 67]. Interestingly in FTLD-TDP-*GRN*lτ brains, a decrease in GS expression has been found. This astrocytic enzyme that converts glutamate into glutamine is frequently deregulated in neurodegenerative diseases presenting with Tau modification [68, 69]. Thus, our results indicate that decrease in GS may underlie glutamate homeostasis alteration, leading to more severe failures in synaptic connectivity and transmission in FTLD-TDP-*GRN*lτ brains. However, why it is limited to cases presenting with point mutations of *GRN* still remains unclear. Beside this, we also found numerous deregulated proteins related to glycolytic metabolism suggesting a critical role for alterations in brain metabolism and energetics in neurodegenerative processes. Therefore, metabolism dysregulation could reflect a more severe pathological state in these brains.

## Conclusions

To conclude, our data reveal that reduction in Tau protein expression is a specific feature of FTLD-TDP cases with *GRN* mutation, suggesting that FTLD-TDP-*GRN*lτ cases could represent a distinct subclass in the current FTLD classification. Moreover, proteomic results clearly demonstrate that in addition to a decrease in Tau protein expression, FTLD-TDP-*GRN*lτ cases also displayed astrocytic and synaptic dysfunctions explaining more severe physiopathological processes. However, we are not currently able to explain this particular feature in part due to the nature of samples, which are post-mortem tissues, and make these dynamic mechanisms investigation complex. If reduced Tau level is a consequence or an actor of deregulations found in these brains remains to be determined and will require development of both in vitro and in vivo models. Finally, further proteomic investigations will also help us to better characterize and understand this particular subclass of FTLD-TDP.

## Additional files

Additional file 1: Figure S1. Preservation of Tau mRNA in FTLD-TDP-*GRN*lτ group. qPCR analysis was done on total Tau mRNA in control and FTLD brain samples. Both 5’UTR (Untranslated Region) and E11-12 (Exons 11–12) primers target regions present in all Tau transcripts. Data were normalized to the mean value of control cases with Large Ribosomal Protein P0 (RPLP0) used as reference gene. Results are expressed as means ± SEM. For statistical analysis the Mann–Whitney test was used (*ns* non significant), *n* = 5–10/group. SEM: standard error of the mean. (TIF 176 kb) Additional file 2: Figure S2. Conservation of several proteins among the different FTLD subclasses. (a) Western blot analysis of NSE (Neuron Specific Enolase), Aconitase, Histone H3 and Heavy (NF-H) Neurofilaments protein level in control and FTLD-U brain samples. Are shown representative data from FTLD-TDP-*GRN*lτ (*n* = 8), FTLD-TDP*-C9ORF72* (*n* = 10), sporadic FTLD-TDP (*n* = 8), sporadic FTLD-FUS (*n* = 5) and control brains (*n* = 8). (b) Protein levels were quantified and normalized to a pool containing same protein amount of each control used in this study. Actin was used as loading control. Results are expressed as means ± SEM. For statistical analysis the Kruskal-Wallis test was used (*ns* non significant). SEM: standard error of the mean. (TIF 223 kb) Additional file 3: Figure S3. Reduction of Tau protein expression does not result from greater post-mortem delay, aberrant RIN or cortical atrophy in FTLD-TDP-*GRN*lτ brains. (a) Fixed hemibrain weight, (b) post-mortem delay and (c) RIN (RNA Integrity Number) of FTLD-TDP-*GRN*lτ, FTLD-TDP-*C9ORF72*, sporadic FTLD-TDP, sporadic FTLD-FUS and control brains. Results are expressed as means ± SEM. For statistical analysis the Kruskal-Wallis test was used (**p* < 0.05; *ns* non significant). *a.u* arbitrary unit, SEM: standard error of the mean. (TIF 164 kb)
