# Supplementary figures and images for: Reduced Tau protein expression is associated with frontotemporal degeneration with progranulin mutation
Source: Acta Neuropathol Commun. 2016 Jul 19;4:74. doi: 10.1186/s40478-016-0345-0 (PMC4952067; doi:10.1186/s40478-016-0345-0)

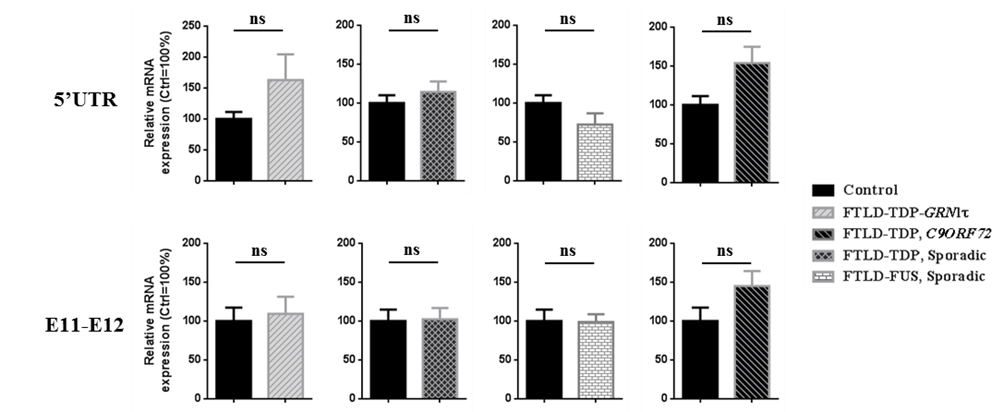

Supplement: Additional file 1: Figure S1. — Preservation of Tau mRNA in FTLD-TDP-GRNlτ group. qPCR analysis was done on total Tau mRNA in control and FTLD brain samples. Both 5’UTR (Untranslated Region) and E11-12 (Exons 11–12) primers target regions present in all Tau transcripts. Data were normalized to the mean value of control cases with Large Ribosomal Protein P0 (RPLP0) used as reference gene. Results are expressed as means ± SEM. For statistical analysis the Mann–Whitney test was used (ns non significant), n = 5–10/group. SEM: standard error of the mean. (TIF 176 kb) [file 40478_2016_345_MOESM1_ESM.tif]

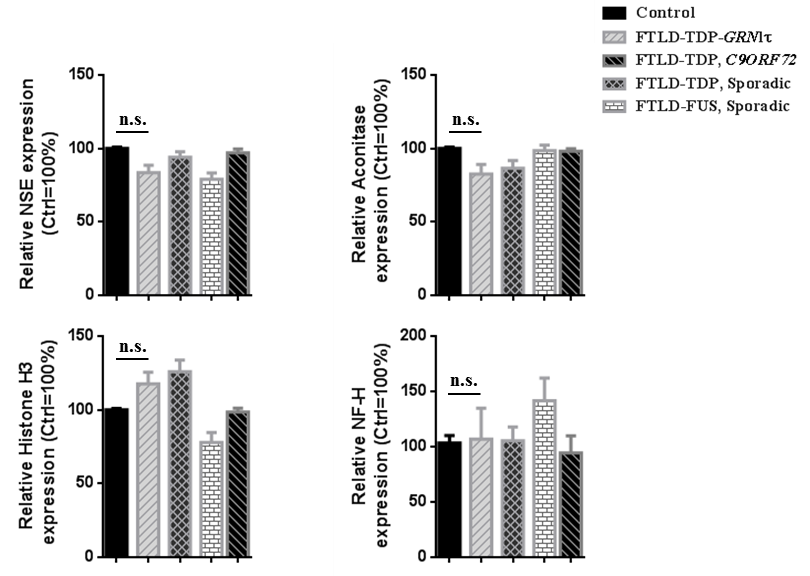

Supplement: Additional file 2: Figure S2. — Conservation of several proteins among the different FTLD subclasses. (a) Western blot analysis of NSE (Neuron Specific Enolase), Aconitase, Histone H3 and Heavy (NF-H) Neurofilaments protein level in control and FTLD-U brain samples. Are shown representative data from FTLD-TDP-GRNlτ (n = 8), FTLD-TDP-C9ORF72 (n = 10), sporadic FTLD-TDP (n = 8), sporadic FTLD-FUS (n = 5) and control brains (n = 8). (b) Protein levels were quantified and normalized to a pool containing same protein amount of each control used in this study. Actin was used as loading control. Results are expressed as means ± SEM. For statistical analysis the Kruskal-Wallis test was used (ns non significant). SEM: standard error of the mean. (TIF 223 kb) [file 40478_2016_345_MOESM2_ESM.tif]

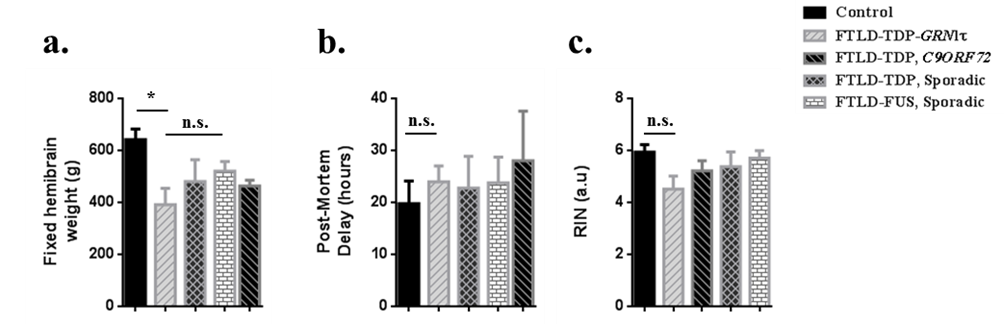

Supplement: Additional file 3: Figure S3. — Reduction of Tau protein expression does not result from greater post-mortem delay, aberrant RIN or cortical atrophy in FTLD-TDP-GRNlτ brains. (a) Fixed hemibrain weight, (b) post-mortem delay and (c) RIN (RNA Integrity Number) of FTLD-TDP-GRNlτ, FTLD-TDP-C9ORF72, sporadic FTLD-TDP, sporadic FTLD-FUS and control brains. Results are expressed as means ± SEM. For statistical analysis the Kruskal-Wallis test was used (*p < 0.05; ns non significant). a.u arbitrary unit, SEM: standard error of the mean. (TIF 164 kb) [file 40478_2016_345_MOESM3_ESM.tif]
